# [Retracted] Effect of Etiasa on the expression of matrix metalloproteinase‑2 and tumor necrosis factor‑α in a rat model of ulcerative colitis

**DOI:** 10.3892/mmr.2025.13513

**Published:** 2025-04-02

**Authors:** Jing-Wei Mao, Hai-Ying Tang, Xiao-Yan Tan, Ying-De Wang

Mol Med Rep 6: 996–1000, 2012; DOI: 10.3892/mmr.2012.1021

Following the publication of this paper, it was drawn to the Editors’ attention by a concerned reader that, in comparing the agarose gels featured in Figs. 1 and 2 on p. 998, two pairs of lanes for the TNF-α data featured for the ‘on day 14/on day 35’ and ‘on day 21/on day 56’ experiments were strikingly similar, although the lanes were featured in reverse orientations relative to each other. Furthermore, MMP-2 protein bands featured in Fig. 2 in the ‘E’ and ‘C’ lanes of the ‘on day 56’ experiments were also strikingly similar in appearance. Finally, concerning the immunofluorescence experiments shown in Figs. 3 and 4, one pair of data panels in each of the figures was found to be overlapping, and moreover, some of the same data panels also appeared in a figure in another paper written by the same research group that was published in the journal *Gastroenterology Research and Practice* at around the same time.

In view of the apparent anomalies associated with the data in Figs. 1 and 2, and the re-use of certain of the data in Figs. 3 and 4, the Editor of *Molecular Medicine Reports* has decided that this paper should be retracted from the Journal on the grounds of a lack of confidence in the presented data. The authors were asked for an explanation to account for these concerns, but the Editorial Office did not receive a reply. The Editor apologizes to the readership for any inconvenience caused.

